# Abdominal Aortic Thrombus Segmentation in Postoperative Computed Tomography Angiography Images Using Bi-Directional Convolutional Long Short-Term Memory Architecture

**DOI:** 10.3390/s23010175

**Published:** 2022-12-24

**Authors:** Younhyun Jung, Suhyeon Kim, Jihu Kim, Byunghoon Hwang, Sungmin Lee, Eun Young Kim, Jeong Ho Kim, Hyoseok Hwang

**Affiliations:** 1 School of Computing, Gachon University, Seongnam 13120, Republic of Korea; 2 Department of Software Convergence, Kyung Hee University, Yongin 17104, Republic of Korea; 3 Department of Radiology, Gil Medical Center, Gachon University, Incheon 21565, Republic of Korea

**Keywords:** abdominal aortic aneurysm, medical image segmentation, computed tomography angiography imaging, mask region-based convolutional neural network, bi-directional convolutional long short-term memory

## Abstract

Abdominal aortic aneurysm (AAA) is a fatal clinical condition with high mortality. Computed tomography angiography (CTA) imaging is the preferred minimally invasive modality for the long-term postoperative observation of AAA. Accurate segmentation of the thrombus region of interest (ROI) in a postoperative CTA image volume is essential for quantitative assessment and rapid clinical decision making by clinicians. Few investigators have proposed the adoption of convolutional neural networks (CNN). Although these methods demonstrated the potential of CNN architectures by automating the thrombus ROI segmentation, the segmentation performance can be further improved. The existing methods performed the segmentation process independently per 2D image and were incapable of using adjacent images, which could be useful for the robust segmentation of thrombus ROIs. In this work, we propose a thrombus ROI segmentation method to utilize not only the spatial features of a target image, but also the volumetric coherence available from adjacent images. We newly adopted a recurrent neural network, bi-directional convolutional long short-term memory (Bi-CLSTM) architecture, which can learn coherence between a sequence of data. This coherence learning capability can be useful for challenging situations, for example, when the target image exhibits inherent postoperative artifacts and noises, the inclusion of adjacent images would facilitate learning more robust features for thrombus ROI segmentation. We demonstrate the segmentation capability of our Bi-CLSTM-based method with a comparison of the existing 2D-based thrombus ROI segmentation counterpart as well as other established 2D- and 3D-based alternatives. Our comparison is based on a large-scale clinical dataset of 60 patient studies (i.e., 60 CTA image volumes). The results suggest the superior segmentation performance of our Bi–CLSTM-based method by achieving the highest scores of the evaluation metrics, e.g., our Bi-CLSTM results were 0.0331 higher on total overlap and 0.0331 lower on false negative when compared to 2D U-net++ as the second-best.

## 1. Introduction

Abdominal aortic aneurysm (AAA) is a life-threatening clinical condition that needs long-term follow-up care and management. AAA is characterized as the abnormal focal dilation of the aorta that exceeds its nomotopic diameter by more than 50% [1]. If left untreated, AAA may gradually expand over time and rupture, resulting in a high mortality rate [2,3,4]. Endovascular aneurysm repair (EVAR) is a dominant treatment approach for AAA, due to its low rate of perioperative mortality and the short period of hospitalization [5,6]. This minimally invasive approach conducts the transfemoral insertion and deployment of a stent graft using a catheter, which isolates the damaged aneurysm wall from the blood circulation. In favorable cases, the thrombus of the isolated region continues to shrink and eventually disappears. Follow-up observation after EVAR, however, is generally required at least yearly due to potential complications, such as endoleaks. These complications, e.g., due to graft material damage or defect, may introduce a recurrent blood leakage toward the isolated thrombus, which potentially requires reintervention to prevent the aortic rupture. In fact, the reintervention rate of EVAR cannot be underestimated, e.g., with a 6-year follow-up showing 29.6% [7].

Computed tomography angiography (CTA) imaging has been the preferred minimally invasive data modality for the follow-up observation of AAA that typically does not cause any noticeable physical symptoms. The use of postoperative CTA imaging allows for the quantitative assessment in the evolution of AAA by imaging specialists. The quantitative assessment should involve the detection of the thrombus region of interest (ROI) and the precise measurement of its morphologic properties, including maximum diameter, volume, and shapes, which, when manually performed by humans, is time-consuming and implies inter- and intra-observer variability [8].

**Figure 1:**
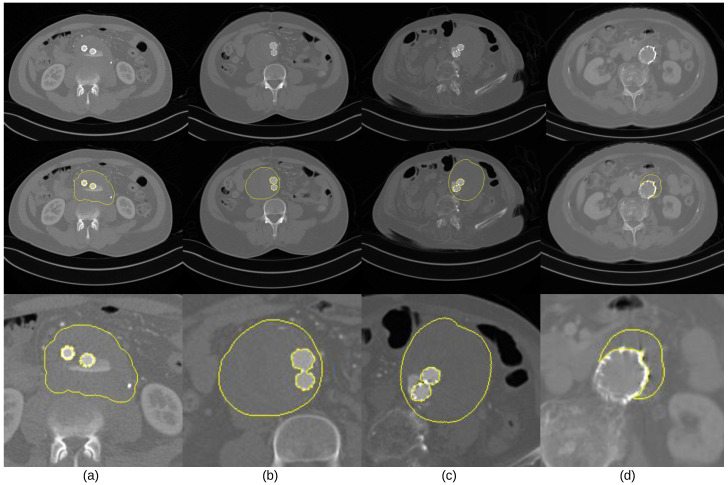
Thrombus ROIs of a CTA image volume. The first row shows four images from different patients, the second row is the thrombus ROIs (annotated by yellow color), and the third row is a focused view of the thrombus ROIs.

To facilitate the quantitative evaluation of thrombus ROIs, computerized segmentation of thrombus ROIs in postoperative CTA imaging was actively investigated [8,9,10]. Traditional segmentation methods are based on semi-automated algorithms using low-level intensity features, such as graph cuts [9], level sets [8], and active models [10]. These segmentation methods, however, are vulnerable to the inherent characteristics in postoperative CTA imaging, which is illustrated in Figure 1 and described below:

- The intensity values of thrombus ROIs tend to overlap with those of adjacent tissues and organs (Figure 1a);
- The geometric shape of thrombus ROIs tends to be irregular, and its position could appear across any part of the abdominal aortic passway (Figure 1b,c);
- Parts of ROIs tend to be occluded by metal artifacts introduced by stent grafts. (Figure 1c,d).

In addition, the traditional segmentation methods often involve user interaction and rely on prior knowledge. The segmentation performance was highly reliant on the careful adjustment of multiple parameters, which affects the robustness for use in clinical settings.

Recent advances in deep learning research using medical imaging continue to address some automation, parameter tuning, user interaction, clinical robustness, and applicability [11,12]. Few investigators have proposed convolutional neural network (CNN)-based methods for automated segmentation of thrombus ROIs [13,14,15,16]. A representative work by López-Linares et al. [13] proposed the adoption of a holistically nested edge detection (HED) network to address the delineation of fuzzy parts (e.g., boundaries) of thrombus ROIs. In these CNN-based methods [13,14,15,16], thrombus ROI segmentation was performed independently per 2D image. These 2D-based methods tended to be vulnerable to challenging situations where an image obviously exhibits the aforementioned characteristics.

In this work, we propose an automated thrombus ROI segmentation method to utilize not only the spatial features of a target image, but also the volumetric coherence among adjacent images. We propose the adoption of a recurrent neural network, bi-directional convolutional long short-term memory (Bi-CLSTM) [17], which can learn coherence between a sequence of data. Bi-CLSTM has the learning capability to remember previous information and reinforce the learning with current information by allowing the previous output to be used as a current input. This coherence learning capability can be useful for challenging situations, for example, where a target image is severe with inherent postoperative artifacts and noises and may need the adjacent images to learn more robust features for thrombus ROIs. For the spatial feature extraction, we use mask region-based CNN (Mask R-CNN) as suggested by Hwang et al. [18]. We experiment the segmentation capability and algorithmic property of our Bi-CLSTM-based method using 60 patient studies of AAA (i.e., postoperative CTA image volumes), which is the largest postoperative AAA dataset to our knowledge. We validate our method by comparing with the existing 2D-based thrombus ROI segmentation counterpart [13] as well as established 2D- and 3D-based alternatives [19,20,21] using five representative evaluation metrics.

The remainder of this paper is described as follows. In Section 2, we discuss the related work for thrombus ROI segmentation. Section 3 describes our proposed method with the experimental settings and datasets in detail. We present and discuss the experiment results and comparisons in Section 4 and Section 5. Finally, concluding remarks are presented in Section 6.

## 2. Related work

### 2.1. Segmentation Methods for Thrombus ROIs in CTA Image Volumes

The traditional methods [8,9,10,22] use prior knowledge, such as geometry constraints and user interaction as a supplement for segmenting thrombus ROIs from CTA image volumes. Given an initial segmentation of the aortic regions by humans, the method proposed by Freiman et al. [9] started to segment thrombus ROIs using an intensity-based graph min-cut algorithm and iteratively refined the segmentation result based on geometry constraints. Lee et al. [8] investigated the use of a 3D graph-cut algorithm to reduce user interaction. In order to improve segmentation accuracy, enhanced geometry constraints were applied by Lareyre et al. [10] and Lalys et al. [22].

Machine learning- and deep learning-based methods have been proposed for enhanced segmentation of thrombus ROIs. Maiora et al. [23] used a random forest classifier which built intensity-based features to better represent thrombus ROIs. Hong et al. [24] introduced deep belief network (DBN) to use deep features. López-Linares et al. [13] firstly introduced the modified holistically-nested edge detection (HED) CNN network to improve the boundary delineation of thrombus ROIs. The use of well-established Mask R-CNN was also investigated by Hwang et al. [18], where an optimized cost function was developed to reinforce the segmentation results. All these 2D-based methods carried out the segmentation independently for each image and lacked the features commonly available from adjacent images. The aim of this study is to investigate the usefulness of adjacent images in enhancing segmentation performance.

### 2.2. CLSTM for ROI Segmentation

Recurrent neural networks are a type of deep learning architecture that is capable of learning coherence between a sequence of data. The LSTM architecture improved the learning process of the recurrent neural networks by addressing the vanishing gradient problems. LSTM architectures have been demonstrated to show notable performance in handling a large variety of sequential problems, e.g., natural language processing [25], machine translation [26], and time-series forecasting [27]. LSTM was composed of a fully connected layer (FCN) that is a 1D vector multiplication, and it inherently posed a limitation for loss of spatial information if LSTM was applied to image sequences. Xingjian et al. [17] introduced a CLSTM that replaced the FCN with a 2D convolutional operation. The proposed architecture demonstrated the capability in maintaining spatial information and notable enhancement in predicting ROIs. CLSTM is now widely applied in, for example, hyperspectral imageries (HSI) classification [28], mitotic cell detection [29], and liver segmentation [30].

## 3. Bi-CLSTM-Based Segmentation Method for Thrombus ROIs

**Figure 2:**
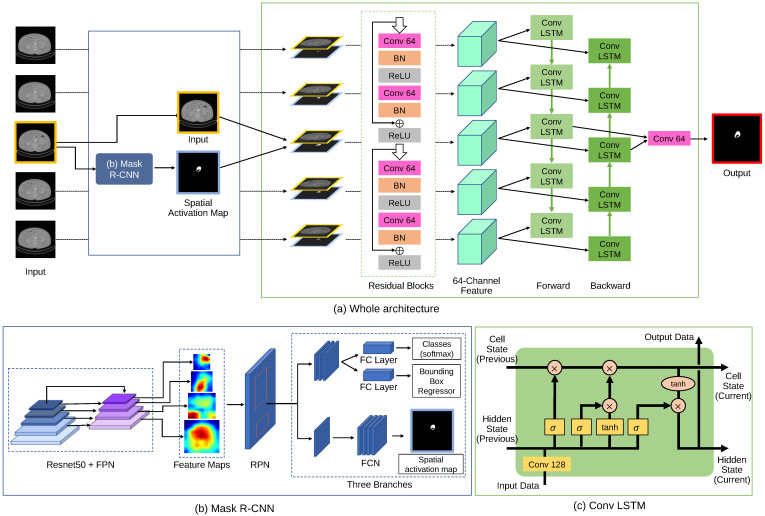
The overview of our Bi-CLSTM-based thrombus ROI segmentation method. Our Bi-CLSTM method inputs a sequence of the five pairs: a pair of the target image and its spatial activation map and the four neighbor pairs. Our Bi-CLSTM-based method performed bi-directional feature integration among the pair sequence. Mask R-CNN was used to extract the spatial activation maps of the individual images.

The whole process of our Bi-CLSTM-based segmentation method for thrombus ROIs in a postoperative CTA image volume is shown in Figure 2. Our method takes as input a pair of the target image and its spatial attention map as input. In addition, neighbor pairs were also put together to learn volumetric coherence between sequences of the pairs. We adopted the concept of Bi-CLSTM [31] by integrating features from the neighbor pairs in a forward and backward manner. We note that CTA image volumes had no direction, and the two different directions may complement each other in the feature learning. For the extraction of the spatial attention map from an image, we adopted the well-established Mask R-CNN [32]. We experimentally set the sequence length of the pairs (*N*) to five for the best segmentation performance but we demonstrated that the different values of the sequence length had no significant influence on the segmentation performance.

### 3.1. Mask R-CNN for Spatial Attention Map

The architecture of Mask R-CNN [32] for the extraction of the spatial attention map is illustrated in Figure 2b. Mask R-CNN consisted of (i) feature pyramid network (FPN) and (ii) region proposal network (RPN). FPN was based on a top-down pathway and used Resnet50 [33] as the backbone. The FPN output of an image was a set of feature maps that were scaled proportionally at various levels. These multi-scale feature maps were used to build high-level semantic feature maps for thrombus ROIs. This pyramid model demonstrated sufficient performance as feature extractors in several general image tasks, such as object detection and segmentation [34]. RPN was designed to generate region (bounding box) proposals. RPN used anchor boxes with three scales and three aspect ratios to address the variation in the sizes of thrombus ROIs. RPN extracted the feature maps from each anchor box and normalized to the same dimension via a bilinear interpolation called RoI Align to accurately locate thrombus ROIs. The region proposals with the normalized feature maps went through each of the three output branches.

Mask R-CNN produced three output branches, including (i) classification, which lets us know whether each region proposal belonged to the foreground class (thrombus ROIs); (ii) regression that estimated bounding box coordinates; and (iii) spatial attention map which produced binary prediction for the class. We only used the spatial attention map for our Bi-CLSTM-based segmentation method. The classification and regression branches used a fully connected (FC) layer with 1024 neurons, and the spatial attention map branch used FCN [35]. We used the loss function for this multi-task learning in [32].

### 3.2. Bi-CLSTM for Volumetric Coherence between an Image Sequence

Our Bi-CLSTM-based segmentation method consisted of two residual blocks and a CLSTM layer (Conv LSTM) as shown in Figure 2a. The residual blocks contained two convolution layers (Conv) with 64 filters and batch normalization (BN) layer, and a single residual connection (ReLU), with the resultant 64-channel feature maps representing the input sequence. The feature map sequence was then transferred to the Conv LSTM. We configured it in the same way as in [17]. It performed a 2D convolutional operation with 128 filters to the input sequence and then mapped the convolutionized sequence to the hidden state, with an output through several gate operations. (see Figure 2c). Our Bi-CLSTM-based segmentation method had two CLSTM operations, each for the forward and the opposite direction. We fused the feature maps from both directions and finally included a Conv with 64 filters to predict thrombus ROI segmentation. We used focal loss [36] to solve the problem of class imbalance between foreground ROIs and backgrounds by assigning the lower weight to the larger background.

### 3.3. Experiment Settings

We built an evaluation dataset of 60 patient studies who went through EVAR operation for the treatment of AAA (i.e., 60 postoperative CTA image volumes). Each patient study had a thrombus ROI in the abdominal aorta. To the best of our knowledge, our evaluation dataset is the largest in the number of patient studies. We observed that the geometrical structure and position of thrombus ROIs exhibited considerable variability in the evaluation dataset, and the noises and artifacts caused by postoperative stent grafts were dispersed randomly (see Figure 1). We believe that our evaluation dataset is obviously suitable for validating the robustness of our method to AAA variability.

The evaluation dataset was acquired by Gachon University Gill Hospital, Republic of Korea, and had been collected for 9 years from 2012 to 2020. The age range of the evaluation dataset is from 51 to 88; this age range includes the elderly population over 65 in which AAA usually occurs [37]. A total of 46 male patients were included along with 14 females; the 3.28:1 ratio was similar to statistics available from the United States (4:1) [38]. Five distinct scanner models from Siemens were used to collect the evaluation dataset: Somatom Definition Edge, Somatom Definition Flash, Somatom Force, Somatom Emotion Duo, and Sensation 16. The patient studies were scanned in the feet-first-supine position, focusing on the chest or abdomen. They varied in pixel spacing from 0.6152 to 0.9766 and the image resolution was 512 × 512 in axial view.

The ground truth (GT) segmentation masks of thrombus ROIs were manually annotated by a senior imaging specialist with more than 10 years of clinical experience. The reliance on the sole annotator may imply that inter and intra-observer variability was present in the evaluation dataset. We generated the ground truth of 2D bounding boxes by fitting rectangles to segmentation masks. We excluded the images from each patient study, where thrombus ROIs did not exist; in total, the number of images was 1637 for use in our evaluation. The full contrast range was rescaled to 8 bits between 0 and 255 without window-level adjustment to minimize human intervention. The window-level contrast adjustment transforms images to focus on specific organs and tissues to obtain better segmentation results. However, it may require the image specialist to manually define the optimal window-level depending on patient studies and scanning devices. So, we decided to use the consistent full contrast range. We could leverage the augmented data to train classifiers to increase reliability. In our method, elastic deformation [19] and rotation within 10° are employed.

We compared our method with three 2D-based methods (U-net [19], U-net++ [21], and mHED [13]) and one 3D-based method (3D U-net [20]) to validate the thrombus ROI segmentation performance of our method. Note that mHED was only the method to be used in the thrombus ROI segmentation, and the others were chosen as representative medical image segmentation methods. For the numerical comparisons, we used five representative evaluation metrics as suggested in [13], which included total overlap (TO), dice coefficient (Dice), and Jaccard index (Jaccard) for overlapping performance and false negative rate (FN) and false positive rate (FP) for overlapping error. We then conducted an ablation study to demonstrate the segmentation capability of our Bi-CLSTM-based method to use volumetric coherence among the adjacent images.

We used a workstation computer with an Intel Core E5-2620 central processing unit at a clock speed of 2.10 GHz and an Nvidia TITAN RTX graphic card for all experiments and pre-processing procedures. Our implementation was based on Pytorch with the Torchvision library [39]. We used stochastic gradient descent optimizer with the learning rate of 0.0001 for Mask R-CNN and 0.005 for Bi-CLSTM. The Adam optimizer with the learning rate of 0.0001 was applied for U-net, mHED, U-net++, and 3D U-net. We conducted 4-fold cross validation to reduce the chance of a biased testing set and provide robustness to evaluation results. Our evaluation dataset was divided by patient study units (i.e., image volumes). For each fold, 45 patient studies were used as a training set, and the other 15 patient studies were as a testing set. We rotated this process 4 times to cover all 60 patient studies for the testing sets.

## 4. Results

### 4.1. Comparison of Our Bi-CLSTM Method with Different 2D and 3D-Based CNN Methods for Thrombus ROI Segmentation

In Table 1, we show the segmentation comparison results of our Bi-CLSTM method with three 2D-based and one 3D-based method [13,19,20,21] for thrombus ROIs. The results show that our Bi-CLTM method outperformed all the existing methods across all the evaluation metrics except for FP where 2D U-net++ method [21] was the best, but the margin with our Bi-CLSTM method by 0.0217 was not obvious. Among the four existing methods, 2D U-net++ produced the overall best performance.

**Figure 3:**
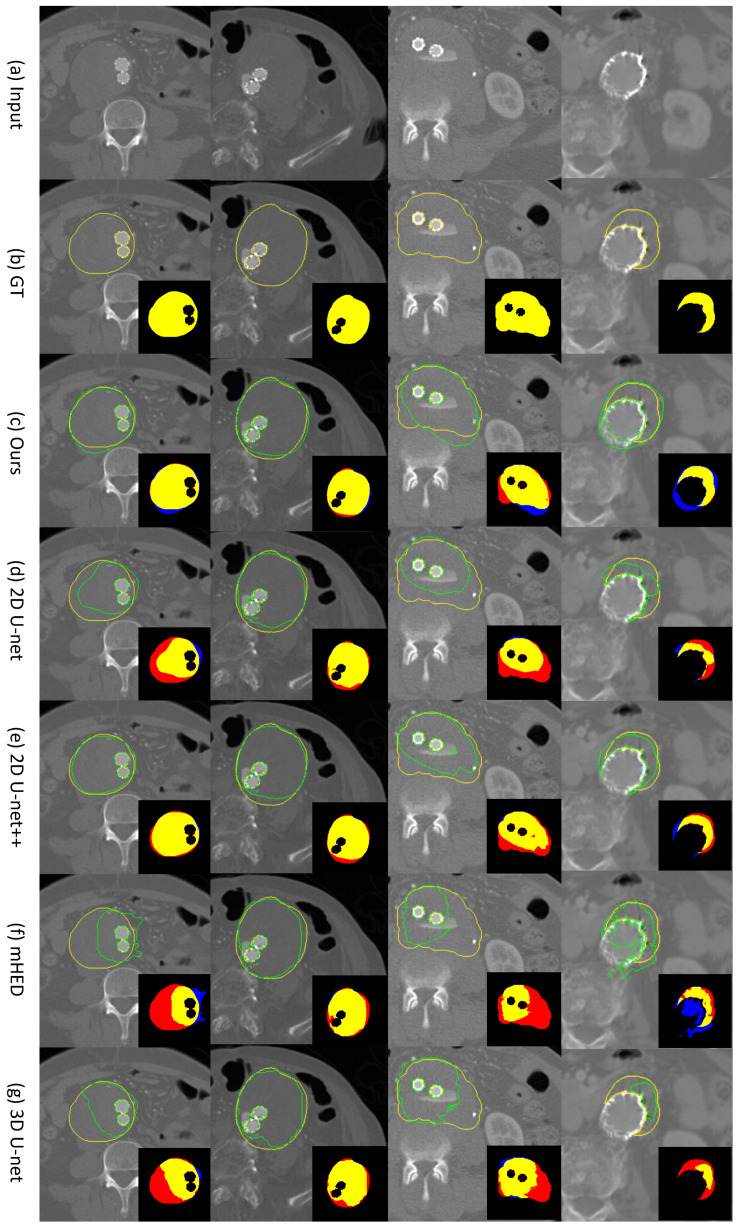
Thrombus ROI segmentation results comparison using four patient studies (each column representing a study). Seven rows represent: (**a**) row image; (**b**) GT mask; (**c**) segmentation result from our Bi-CLSTM method; (**d**) 2D U-net; (**e**) 2D U-net++; (**f**) mHED; (**g**) 3D U-net. In full-scale images, the yellow line denotes the GT; and the green line is the predicted mask from different methods. In cropped insets, the yellow color indicates true positive voxels; the red color is false negative; and the blue color is false positive.

In Figure 3, we show the qualitative visualization results from four patient studies for all five methods [13,19,20,21]. The results showed our Bi-CLSTM method enhanced the prediction capability in segmenting voxels belonging to the thrombus ROIs, when compared to the four existing methods. We note that the enhancement was obvious in the areas that were difficult to be differentiated from the neighboring tissues and organs due to the similarity in low-level features. It is observed that our Bi-CLSTM method tended to perform over-segmentation (see the region with blue color), whereas the other existing methods exhibited an under-segmentation tendency (see the region with red color), which was consistent with the quantitative findings from FP scores in Table 1.

### 4.2. Ablation Study of Our Bi-CLSTM-Based Method

Our Bi-CLSTM-based method relied on a 2D-based CNN backbone to extract the spatial attention map of images, which were then used to learn the volumetric coherence of the image sequence. In Table 2, we evaluated the utility of the volumetric coherence by comparing the segmentation results of thrombus ROIs with and without it, i.e., the spatial attention map from the 2D-based CNN backbones can be a segmentation result. For generalization, we used four different 2D-based CNN backbones, including our Mask R-CNN and the 2D U-net [19], 2D U-net++ [21], and mHed [13]. The results show that all four 2D-based CNN backbones could benefit from the use of the volumetric coherence, improving the evaluation metrics in large margins, e.g., 0.1024 of TO on mHED. Our Mask R-CNN and mHED backbones resulted in the segmentation enhancement from all five evaluation metrics. On the other hand, 2D U-net++ backbone showed degeneration from the FP metric, but the margin was not obvious, and the gains from the other four metrics were much greater. Similarly, 2D U-net did not provide the segmentation enhancement from the Dice, Jaccard, and FP metrics. We used Mask R-CNN as the default backbone of our method because Mask R-CNN showed the greatest enhancement.

In Table 3, we show the influence of the image sequence length (*N*) in our Bi-CLSTM-based method on the thrombus ROI segmentation results. We experimented with the three different lengths of image sequences ranging from 3, 5, to 7. The results show that our Bi-CLSTM-based method with the sequence of 5 images had the highest for the Dice, Jaccard, and FP metrics; the TO and FN metrics were high with the longest sequence of 7 images. We, however, note that there were no significant differences among all three sequence lengths. We chose 5 as the default sequence length due to its advantage in lower memory consumption when compared to the longest length of 7.

## 5. Discussion and Future Works

The results demonstrate the capability of our Bi-CLSTM-based method to precisely segment thrombus ROIs in post-operative CTA image volumes, where different artifacts and noises commonly appear. The comparative validation with the large-scale patient studies suggests the robustness and utility of our Bi-CLSTM-based method over the four existing counterparts [13,19,20,21].

We attribute our superior segmentation performance to the novel use of Bi-CLSTM which was designed to take into account the volumetric coherence of image sequences. The ROI segmentation of an image could be refined with additional features learned from adjacent images. The value of the volumetric coherence was experimentally validated from the comparison results with the 2D-based segmentation methods [13,19,21], where the thrombus ROIs were segmented independently per image, i.e., without the volumetric coherence. Our BI-CLSTM-based method outperformed all 2D-based counterparts in the evaluation metrics (see Table 1). In addition, we visually observed that the 2D-based methods failed the boundary parts of thrombus ROIs to be precisely segmented due to the neighboring tissues sharing similar low-level features, such as intensity values (see Figure 3).

We demonstrated the enhanced segmentation capability of our Bi-CLSTM-based method over the 3D-based counterpart [20] by showing its superior performance from the evaluation metrics (see Table 1). It could suggest that our Bi-CLSTM-based method can be more effective to preserve the volumetric coherence by explicitly combining the features within adjacent images, when compared to the 3D-based counterpart, implicitly extracting the volumetric coherence through a 3D convolution operation. We analyzed that one of the dominant advantages of the proposed method over 3D-based methods is that our approach utilizes information from adjacent slices, whereas 3D u-net also uses information from slices farther away.

We valued the over-segmentation tendency of our Bi-CLSTM-based method, instead of the under-segmentation tendency from the existing methods [13,19,20,21] (see Table 1 and Figure 3). From the clinical point of view, it is ideal to avoid both specificity (FP) and sensitivity (FN) for patient care and management, but if not available, having lower specificity is preferred over sensitivity [40]. It is due to the fact that FN could have deadly consequences, but FN could be resolved later by trained clinicians.

The current results may imply that our Bi-CLSTM-based segmentation method is limited in clinical generalization and robustness because we validated it only with a single medical institution. Medical institutions vary in, for example, imaging scanner manufacturers, image acquisition protocols, and clinical routines. These variations have a major impact on the quality of postoperative CTA image volumes and the segmentation performance for thrombus ROIs. As future work, we plan to achieve a certain level of clinical generalization and robustness by validating our method with multi-site medical institutions.

Our results (see Table 2) demonstrate the adaptability of our Bi-CLSTM-based method to different CNN backbones for the extraction of the spatial attention maps. Mask R-CNN and mHED following Bi-CLSTM showed better performance in all evaluation metrics. However, only total overlap (TO) and the false negative (FN) were improved in the case of U-net-based backbones. As such, our Bi-CLSTM-based method is not limited to a particular CNN backbone, but we experimentally chose Mask R-CNN as the default due to the superior segmentation performance. The investigation to use other new CNN backbones could be an interesting future work in order to further improve the segmentation performance.

## 6. Conclusions

We proposed a noise-robust Bi-CLSTM-based segmentation method for thrombus ROIs in postoperative CTA image volumes. Our Bi-CLSTM-based segmentation method approximates the thrombus ROIs of a target image and then refines the challenging perturbation parts, e.g., the boundaries, using the volumetric coherence of the adjacent images, thereby improving overall segmentation performance. The thorough validations with a large-scale clinical dataset of 60 patient studies suggest that our method is superior to the existing 2D-based and 3D-based counterparts [13,19,20,21]. We suggest that our work can be a useful baseline for the quantitative assessment of postoperative AAA clinical conditions.

## Figures and Tables

**Table 1:** Thrombus ROI segmentation result comparison of our Bi-CLSTM method with three 2D-based [13,19,21] and one 3D-based approach [20] using the five evaluation metrics.

	TO ↑	Dice ↑	Jaccard ↑	FN ↓	FP ↓
Our Bi-CLSTM	**0.8931**	**0.8730**	**0.7809**	**0.1069**	0.1358
2D Segmentation Approaches					
2D U-net	0.8297	0.8375	0.7334	0.1703	0.1277
2D U-net++	0.8600	0.8655	0.7703	0.1400	**0.1141**
mHED	0.6210	0.6488	0.4949	0.3790	0.2610
3D Segmentation Approaches					
3D U-net	0.7545	0.7145	0.5768	0.2455	0.2687

**Table 2:** Thrombus ROI segmentation results comparison among four 2D-based CNN backbones for use in our Bi-CLSTM method.

		TO ↑	Dice ↑	Jaccard ↑	FN ↓	FP ↓
Mask R-CNN (ours)	Without Bi-CLSTM	0.8762	0.8636	0.7666	0.1238	0.1383
	With Bi-CLSTM	**0.8931**	**0.8730**	**0.7809**	**0.1069**	0.1358
2D U-net	Without Bi-CLSTM	0.8297	0.8375	0.7334	0.1703	0.1277
	With Bi-CLSTM	0.8399	0.8319	0.7253	0.1601	0.1483
2D U-net++	Without Bi-CLSTM	0.8600	0.8655	0.7703	0.1400	**0.1141**
	With Bi-CLSTM	0.8856	0.8570	0.7563	0.1144	0.1567
mHED	Without Bi-CLSTM	0.6210	0.6488	0.4949	0.3790	0.2610
	With Bi-CLSTM	0.7234	0.7110	0.5671	0.2766	0.2459

**Table 3:** Thrombus ROI segmentation results from three different lengths (N) of the image sequence for our Bi-CLSTM-based method.

Image Sequence Length of Bi-CLSTM	TO	Dice	Jaccard	FN	FP
3	0.8921	0.8723	0.7798	0.1079	0.1365
5	0.8931	**0.8730**	**0.7809**	0.1069	**0.1358**
7	**0.8937**	0.8712	0.7782	**0.1063**	0.1390

## Data Availability

The data are not publicly available due to privacy restrictions.

## References

[1] Wang L.J., Prabhakar A.M., Kwolek C.J. (2018). Current status of the treatment of infrarenal abdominal aortic aneurysms. Cardiovasc. Diagn. Ther..

[2] Acosta S., Ögren M., Bengtsson H., Bergqvist D., Lindblad B., Zdanowski Z. (2006). Increasing incidence of ruptured abdominal aortic aneurysm: A population-based study. J. Vasc. Surg..

[3] Vorp D.A., Geest J.P.V. (2005). Biomechanical determinants of abdominal aortic aneurysm rupture. Arterioscler. Thromb. Vasc. Biol..

[4] Tuns F., Iurcut A., Chirilean I., Crivii C., Damian A. (2013). Comparative Bibliographic Study Regarding the Collaterals of Ascending Aorta and Aortic Cross in Humans, Swine and Equine. Sci. Work Ser. C Vet. Med..

[5] National Institute for Health and Care Excellence (2020). Abdominal Aortic Aneurysm: Diagnosis and Management.

[6] Varkevisser R.R., O’Donnell T.F., Swerdlow N.J., Liang P., Li C., Ultee K.H., Pothof A.B., De Guerre L.E., Verhagen H.J., Schermerhorn M.L. (2019). Fenestrated endovascular aneurysm repair is associated with lower perioperative morbidity and mortality compared with open repair for complex abdominal aortic aneurysms. J. Vasc. Surg..

[7] De Bruin J.L., Baas A.F., Buth J., Prinssen M., Verhoeven E.L., Cuypers P.W., van Sambeek M.R., Balm R., Grobbee D.E., Blankensteijn J.D. (2010). Long-term outcome of open or endovascular repair of abdominal aortic aneurysm. N. Engl. J. Med..

[8] Lee K., Johnson R.K., Yin Y., Wahle A., Olszewski M.E., Scholz T.D., Sonka M. (2010). Three-dimensional thrombus segmentation in abdominal aortic aneurysms using graph search based on a triangular mesh. Comput. Biol. Med..

[9] Freiman M., Esses S.J., Joskowicz L., Sosna J. An iterative model-constrained graph-cut algorithm for abdominal aortic aneurysm thrombus segmentation. Proceedings of the 2010 IEEE International Symposium on Biomedical Imaging: From Nano to Macro.

[10] Lareyre F., Adam C., Carrier M., Dommerc C., Mialhe C., Raffort J. (2019). A fully automated pipeline for mining abdominal aortic aneurysm using image segmentation. Sci. Rep..

[11] Litjens G., Kooi T., Bejnordi B.E., Setio A.A.A., Ciompi F., Ghafoorian M., Van Der Laak J.A., Van Ginneken B., Sánchez C.I. (2017). A survey on deep learning in medical image analysis. Med. Image Anal..

[12] Seo H., Huang C., Bassenne M., Xiao R., Xing L. (2019). Modified U-Net (mU-Net) with incorporation of object-dependent high level features for improved liver and liver-tumor segmentation in CT images. IEEE Trans. Med. Imaging.

[13] López-Linares K., Aranjuelo N., Kabongo L., Maclair G., Lete N., Ceresa M., García-Familiar A., Macía I., Ballester M.A.G. (2018). Fully automatic detection and segmentation of abdominal aortic thrombus in post-operative CTA images using deep convolutional neural networks. Med. Image Anal..

[14] Lu J.T., Brooks R., Hahn S., Chen J., Buch V., Kotecha G., Andriole K.P., Ghoshhajra B., Pinto J., Vozila P. (2019). DeepAAA: Clinically applicable and generalizable detection of abdominal aortic aneurysm using deep learning. Proceedings of the International Conference on Medical Image Computing and Computer-Assisted Intervention.

[15] Caradu C., Spampinato B., Vrancianu A.M., Bérard X., Ducasse E. (2021). Fully automatic volume segmentation of infrarenal abdominal aortic aneurysm computed tomography images with deep learning approaches versus physician controlled manual segmentation. J. Vasc. Surg..

[16] Lareyre F., Adam C., Carrier M., Raffort J. (2021). Automated segmentation of the human abdominal vascular system using a hybrid approach combining expert system and supervised deep learning. J. Clin. Med..

[17] Shi X., Chen Z., Wang H., Yeung D.Y., Wong W.K., Woo W.c. (2015). Convolutional LSTM network: A machine learning approach for precipitation nowcasting. Adv. Neural Inf. Process. Syst..

[18] Hwang B., Kim J., Lee S., Kim E., Kim J., Jung Y., Hwang H. (2022). Automatic Detection and Segmentation of Thrombi in Abdominal Aortic Aneurysms Using a Mask Region-Based Convolutional Neural Network with Optimized Loss Functions. Sensors.

[19] Ronneberger O., Fischer P., Brox T. (2015). U-net: Convolutional networks for biomedical image segmentation. Proceedings of the International Conference on Medical Image Computing and Computer-Assisted Intervention.

[20] Çiçek Ö., Abdulkadir A., Lienkamp S.S., Brox T., Ronneberger O. (2016). 3D U-Net: Learning dense volumetric segmentation from sparse annotation. Proceedings of the International Conference on Medical Image Computing and Computer-Assisted Intervention.

[21] Zhou Z., Rahman Siddiquee M.M., Tajbakhsh N., Liang J. (2018). Unet++: A nested u-net architecture for medical image segmentation. Deep Learning in Medical Image Analysis and Multimodal Learning for Clinical Decision Support.

[22] Lalys F., Yan V., Kaladji A., Lucas A., Esneault S. (2017). Generic thrombus segmentation from pre-and post-operative CTA. Int. J. Comput. Assist. Radiol. Surg..

[23] Maiora J., Graña M. Abdominal CTA image analisys through active learning and decision random forests: Aplication to AAA segmentation. Proceedings of the 2012 International Joint Conference on Neural Networks (IJCNN).

[24] Hong H.A., Sheikh U. Automatic detection, segmentation and classification of abdominal aortic aneurysm using deep learning. Proceedings of the 2016 IEEE 12th International Colloquium on Signal Processing & Its Applications (CSPA).

[25] Ali F., El-Sappagh S., Kwak D. (2019). Fuzzy ontology and LSTM-based text mining: A transportation network monitoring system for assisting travel. Sensors.

[26] Crivellari A., Beinat E. (2020). Trace2trace—A feasibility study on neural machine translation applied to human motion trajectories. Sensors.

[27] Sagheer A., Hamdoun H., Youness H. (2021). Deep LSTM-based transfer learning approach for coherent forecasts in hierarchical time series. Sensors.

[28] Seydgar M., Alizadeh Naeini A., Zhang M., Li W., Satari M. (2019). 3-D convolution-recurrent networks for spectral-spatial classification of hyperspectral images. Remote Sens..

[29] Kitrungrotsakul T., Han X.H., Iwamoto Y., Takemoto S., Yokota H., Ipponjima S., Nemoto T., Xiong W., Chen Y.W. (2019). A cascade of 2.5 D CNN and bidirectional CLSTM network for mitotic cell detection in 4D microscopy image. IEEE/ACM Trans. Comput. Biol. Bioinform..

[30] Jeong J.G., Choi S., Kim Y.J., Lee W.S., Kim K.G. (2022). Deep 3D attention CLSTM U-Net based automated liver segmentation and volumetry for the liver transplantation in abdominal CT volumes. Sci. Rep..

[31] Graves A., Mohamed A.R., Hinton G. Speech recognition with deep recurrent neural networks. Proceedings of the 2013 IEEE International Conference on Acoustics, Speech and Signal Processing.

[32] He K., Gkioxari G., Dollár P., Girshick R. Mask r-cnn. Proceedings of the IEEE International Conference on Computer Vision.

[33] He K., Zhang X., Ren S., Sun J. Deep residual learning for image recognition. Proceedings of the IEEE Conference on Computer Vision and Pattern Recognition.

[34] Lin T.Y., Dollár P., Girshick R., He K., Hariharan B., Belongie S. Feature pyramid networks for object detection. Proceedings of the IEEE Conference on Computer Vision and Pattern Recognition.

[35] Long J., Shelhamer E., Darrell T. Fully convolutional networks for semantic segmentation. Proceedings of the IEEE Conference on Computer Vision and Pattern Recognition.

[36] Lin T.Y., Goyal P., Girshick R., He K., Dollár P. Focal loss for dense object detection. Proceedings of the IEEE International Conference on Computer Vision.

[37] Vardulaki K., Walker N., Day N., Duffy S., Ashton H., Scott R. (2000). Quantifying the risks of hypertension, age, sex and smoking in patients with abdominal aortic aneurysm. J. Br. Surg..

[38] Harthun N.L. (2008). Current issues in the treatment of women with abdominal aortic aneurysm. Gend. Med..

[39] Torchvision. https://pytorch.org/vision/stable/index.html.

[40] Burt T., Button K., Thom H., Noveck R., Munafò M.R. (2017). The Burden of the “False-Negatives” in Clinical Development: Analyses of Current and Alternative Scenarios and Corrective Measures. Clin. Transl. Sci..

